# The Role of Neutrophil-to-Lymphocyte Ratio in Predicting Outcomes of Acute Organophosphorus Poisoning: A Comprehensive Review

**DOI:** 10.7759/cureus.60854

**Published:** 2024-05-22

**Authors:** Utkarsh Pradeep, Anjalee Chiwhane, Sourya Acharya, Sunil Kumar, Varun Daiya, Paschyanti R Kasat, Aman Gupta, Gautam N Bedi

**Affiliations:** 1 Medicine, Jawaharlal Nehru Medical College, Datta Meghe Institute of Higher Education and Research, Wardha, IND; 2 Radiodiagnosis, Jawaharlal Nehru Medical College, Datta Meghe Institute of Higher Education and Research, Wardha, IND

**Keywords:** neutrophil-to-lymphocyte ratio (nlr), risk stratification, inflammation, clinical outcomes, prognostic marker, organophosphorus poisoning

## Abstract

Organophosphorus poisoning (OPP) poses a significant threat to human health, necessitating accurate prognostic markers for timely intervention and improved outcomes. This review evaluates the potential of the neutrophil-to-lymphocyte ratio (NLR) as a prognostic indicator in acute organophosphorus poisoning (AOPP). A comprehensive analysis of existing literature reveals that elevated NLR values correlate with increased severity of poisoning and adverse clinical outcomes, including mortality and morbidity. NLR assessment offers valuable prognostic information beyond traditional markers, aiding risk stratification and guiding clinical decision-making. Integration of NLR into clinical practice holds promise for optimizing patient care through the early identification of high-risk individuals and tailored therapeutic interventions. Further research is needed to validate the utility of NLR in larger patient cohorts and standardize its incorporation into clinical guidelines. Leveraging NLR as a prognostic tool can enhance risk stratification, optimize treatment strategies, and ultimately improve outcomes in AOPP.

## Introduction and background

Organophosphorus (OP) compounds are a class of chemicals commonly used in pesticides, insecticides, and nerve agents. Accidental or intentional exposure to these compounds can lead to organophosphorus poisoning (OPP), which poses a significant threat to human health [1]. The toxicity of OP compounds stems from their ability to inhibit acetylcholinesterase (AChE), resulting in the accumulation of acetylcholine (ACh) at nerve synapses and subsequent overstimulation of cholinergic receptors [2]. Acute organophosphorus poisoning (AOPP) can manifest with a wide range of symptoms, ranging from mild cholinergic symptoms such as nausea and vomiting to severe manifestations including respiratory failure, seizures, and death. Predicting the clinical course and outcomes of acute poisoning is crucial for timely intervention and appropriate management strategies [3]. Early identification of patients at high risk of adverse outcomes can guide healthcare providers in allocating resources effectively and implementing targeted therapies to improve patient outcomes [4].

Neutrophil-to-lymphocyte ratio (NLR) is a simple and readily available marker of systemic inflammation, calculated by dividing the absolute neutrophil count by the absolute lymphocyte count from a routine complete blood count (CBC). Elevated NLR has been implicated as a prognostic indicator in various medical conditions, reflecting an imbalance between pro-inflammatory neutrophils and anti-inflammatory lymphocytes [5]. The purpose of this review is to comprehensively evaluate the role of NLR in predicting outcomes of AOPP. By synthesizing existing literature, we aim to elucidate the potential utility of NLR as a prognostic marker in this clinical setting. Additionally, we will discuss the underlying pathophysiological mechanisms linking inflammation to OP toxicity and explore the implications of NLR assessment in clinical practice.

## Review

Pathophysiology of OPP

Mechanism of Action of OP Compounds

The mechanism by which OP compounds exert their effects involves the inhibition of AChE through phosphorylation of the serine hydroxyl group in the enzyme's active site. This phosphorylation event results in the inactivation of AChE, leading to an accumulation of ACh at nerve synapses and neuromuscular junctions. Consequently, this accumulation triggers a series of cholinergic effects throughout the body [1,6]. The inhibition of AChE is pivotal for the toxic impact of OP compounds on insects and mammals, as it disrupts normal cholinergic signaling pathways, manifesting in a spectrum of symptoms affecting various bodily systems [6]. Moreover, while it is widely acknowledged that organophosphates exert their insecticidal activity by inhibiting cholinesterase activity in the nervous system, definitive proof of this theory remains debated within the scientific community [7]. Extensive research has delved into the precise structural requirements and reactivity of OP compounds necessary for their anticholinesterase activity, providing insights into the intricate mechanisms underpinning their toxic effects on biological systems [7]. Thus, the mechanism of action of OP compounds entails the inhibition of AChE, which disrupts cholinergic neurotransmission and elicits a range of toxic effects in both insects and mammals.

Physiological Effects on the Body

The physiological effects on the body encompass a broad spectrum of responses influenced by factors such as stress, exercise, and environmental stimuli. These effects impact multiple bodily systems, including the musculoskeletal, respiratory, cardiovascular, endocrine, gastrointestinal, and nervous [8]. Stress induces a complex array of physiological responses involving various bodily systems. Activation of the autonomic nervous system, particularly the sympathetic division, initiates the well-known "fight or flight" response, releasing hormones such as adrenaline and cortisol. Prolonged exposure to stress can result in chronic wear and tear on the body, affecting diverse systems and potentially contributing to health conditions like hypertension, heart disease, and inflammation in the circulatory system [8]. Exercise elicits profound physiological changes throughout the body, affecting musculoskeletal, respiratory, cardiovascular, and cognitive systems. The body's oxygen and substrate requirements escalate during physical activity, prompting increased blood flow to active muscles. The cardiovascular system responds by elevating heart rate and stroke volume to meet the augmented oxygen demand. Consistent exercise fosters muscle strengthening, enhances bone density, improves cardiovascular efficiency, and positively influences overall health and wellness [8-10]. Comprehending these physiological effects is paramount for understanding the body's responses to stressors, physical exertion, and environmental cues. It underscores the intricate interplay among various bodily systems in maintaining homeostasis and promoting overall health and well-being.

Time Course of Poisoning and Clinical Manifestations

The time course of organophosphate poisoning and its clinical manifestations can exhibit variability depending on factors such as the specific agent, route of exposure, and quantity encountered. Typically, symptoms manifest within minutes to hours following acute exposure, with onset categorized into acute (within 24 hours), delayed (24 hours to 2 weeks), and late (beyond two weeks) presentations [11]. Clinical manifestations of organohosphate poisoning encompass a broad spectrum of effects stemming from muscarinic and nicotinic actions. Muscarinic effects give rise to symptoms such as increased salivation, tearing, urination, diarrhea, gastrointestinal disturbances, vomiting, and other manifestations affecting various organ systems, including cardiovascular, respiratory, gastrointestinal, genitourinary, ocular, and glandular systems [11,12]. In contrast, nicotinic effects manifest as muscle fasciculations, cramping, weakness, and autonomic symptoms like hypertension, tachycardia, pupil dilation, and pallor. Central nervous system (CNS) involvement encompasses anxiety, emotional instability, restlessness, confusion, ataxia, tremors, seizures, coma, and respiratory arrest [11]. Moreover, organohosphate poisoning can result in three distinct types of paralysis: acute paralysis (type I), intermediate syndrome (type II), and OP-induced delayed polyneuropathy (OPIDP) (type III) [11]. Type I paralysis entails acute neuromuscular junction depolarization, leading to rapid paralysis. Type II paralysis manifests as respiratory distress and paralysis emerging 24-96 hours after acute symptom resolution. OPIDP emerges 2-3 weeks postexposure to substantial organohosphate doses, characterized by distal muscle weakness, with recovery potentially extending up to 12 months [11].

Role of inflammation in OPP

Inflammatory Response to Poisoning

The inflammatory response elicited by poisoning is a multifaceted process involving activating diverse immune cells and the release of pro-inflammatory mediators. In the context of organohosphate poisoning, AChE inhibition prompts an accumulation of ACh, triggering a cholinergic crisis characterized by muscarinic and nicotinic signs [13]. This crisis is accompanied by a vigorous inflammatory reaction, contributing to the onset of respiratory complications and other systemic effects. Systemic inflammatory response syndrome (SIRS) represents an exaggerated defensive reaction of the body to a harmful stressor, encompassing poisoning [14]. Various triggers, including infection, trauma, surgery, acute inflammation, ischemia or reperfusion, or malignancy, can initiate SIRS. It involves the release of acute-phase reactants like C-reactive protein and activating immune cells such as neutrophils and macrophages. In the context of poisoning, SIRS can culminate in end-organ dysfunction and multiple organ failure, which serve as pivotal determinants of mortality [14]. The emergence of SIRS following poisoning is influenced by several factors, including the type and dosage of the toxin, the route of exposure, and the individual's underlying health condition. The inflammatory response to poisoning, particularly in cases of organohosphate poisoning, constitutes a crucial aspect of the body's defense mechanism. However, an exaggerated or uncontrolled inflammatory response can precipitate SIRS, leading to grave consequences such as end-organ dysfunction and mortality.

Neutrophils and Lymphocytes in the Context of Poisoning

Neutrophils and lymphocytes play pivotal roles in the context of poisoning, particularly in AOPP. Neutrophils, integral to the immune system, initiate the initial inflammatory response to toxic insults. In poisoning scenarios, they can be activated and mobilized to the injury site, thus contributing to the inflammatory cascade and subsequent tissue damage [15]. Conversely, lymphocytes, encompassing T and B cells, are crucial for adaptive immunity and help regulate the immune response to toxins. In cases of poisoning, these lymphocytes can be impacted by the toxic insult, potentially leading to alterations in immune function and affecting the body's capacity to mount an effective immune response [15]. As evidenced by the NLR, the equilibrium between neutrophils and lymphocytes has been under scrutiny as a potential prognostic marker in AOPP. Elevated NLR levels may signify a more robust inflammatory response and poorer outcomes in poisoning cases [16-19]. The interaction between neutrophils and lymphocytes holds critical importance in the immune response to poisoning, with the NLR serving as a valuable gauge of inflammatory status and prognosis in AOPP.

Importance of NLR as an Inflammatory Marker

The NLR has emerged as a significant inflammatory marker for predicting outcomes in cases of AOPP [20-22]. OP compounds can incite a robust inflammatory response during acute poisoning episodes, contributing to respiratory complications and other systemic effects [20,22]. While the inhibition of AChE by OPs leads to ACh accumulation and triggers a cholinergic crisis, additional mechanisms beyond AChE inhibition, such as lung edema, tissue destruction, and altered immune responses, can precipitate acute respiratory distress syndrome (ARDS) in severe organohosphate poisoning cases [22]. Research indicates that organohosphate exposure can modulate inflammation by impacting macrophages and other immune cells [22]. For instance, the pro-inflammatory cytokine IL-6 enhances the polarization of alternatively activated macrophages in response to organohosphate poisoning [22]. Studies have demonstrated that the prognostic value of NLR, when measured in patients within 24 hours of admission, is markedly higher in severe poisoning cases compared to milder instances [22]. Moreover, patients necessitating mechanical ventilation exhibited elevated leukocyte and neutrophil counts, increased NLR, and decreased lymphocyte counts [22]. Consequently, NLR and other hematological indices may be early biomarkers of OP exposure-induced inflammation. Nonetheless, it is worth noting that chronic exposure could potentially obscure the degree of intoxication based on NLR levels. NLR stands as a valuable inflammatory marker capable of predicting outcomes in acute organophosphate poisoning cases. A comprehensive understanding of the mechanisms underlying OP-induced inflammation is imperative for devising targeted therapeutic interventions to mitigate the adverse consequences of exposure [21].

Methods for assessing NLR

Laboratory Methods for NLR Determination

**Figure 1:**
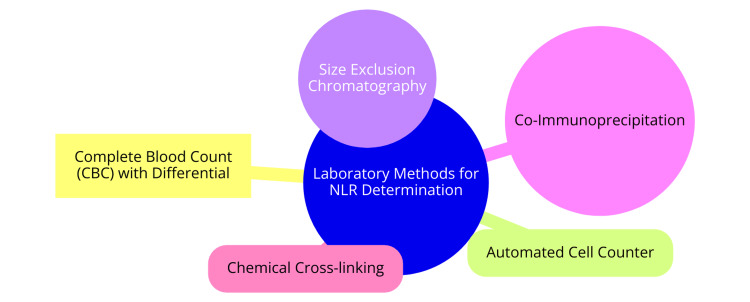
Laboratory methods for NLR determination Image created by Dr. Utkarsh Pradeep

CBC with differential is a standard procedure to obtain absolute counts of neutrophils and lymphocytes. Subsequently, the NLR is computed by dividing the absolute neutrophil count by the absolute lymphocyte count [23]. An automated cell counter, employing mixed technologies, is another method to determine CBC parameters, including absolute neutrophil and lymphocyte counts. From these values, the NLR is calculated [23]. Size exclusion chromatography (SEC) facilitates the separation of native protein lysates into various-sized complexes via fast protein liquid chromatography (FPLC). This method enables the detection of NLR oligomerization, contingent upon the stringency of protein-protein interactions [24]. Co-immunoprecipitation (Co-IP), coupled with SEC or conducted independently, enables antibody-based purification of NLR complexes and associated proteins. Subsequent analysis involves immunoblotting and functional assays such as caspase-1 activity assay [24]. Chemical cross-linking, through covalent bonding of two or more molecules, captures the oligomeric state of NLRs with high sensitivity and stability. This technique has effectively utilized apoptosis-associated speck-like protein with a caspase-recruitment domain (ASC) oligomerization as a readout for NLR/aspartate aminotransferase-to-lymphocyte ratio (ALR) inflammasome activation in response to various PAMPs [24]. Primarily, the NLR is determined via CBC with differential, calculating the ratio of absolute neutrophil to lymphocyte counts. Advanced techniques like SEC, Co-IP, and chemical cross-linking further elucidate the oligomerization and activation of NLRs in inflammasome signaling. Laboratory methods for NLR determination are shown in Figure 1.

Timing of NLR Assessment in Acute Poisoning Cases

The optimal timeframe for evaluating the NLR in cases of AOPP is within 24 hours of admission or initial presentation [25,26]. Several studies have focused on assessing NLR upon admission to the hospital or intensive care unit [25], enabling early risk assessment and guiding timely interventions. NLR values obtained shortly after poisoning offer insights into the initial inflammatory response and oxidative stress, both implicated in the pathogenesis of organophosphate neurotoxicity [26,27]. However, it is essential to acknowledge that the duration between poisoning and hospitalization may vary. In one study, the majority of patients (82.4%) were admitted within six hours of toxic exposure, while a small percentage (12.1%) arrived after 24 hours [28]. Therefore, evaluating NLR within 24 hours of admission, irrespective of the exact poisoning timing, ensures assessment of the inflammatory marker during the early stages of clinical presentation. While NLR assessment is ideally conducted within 24 hours of initial presentation, the precise timing may hinge on laboratory result availability and the patient's clinical trajectory. Consistent NLR evaluation during this critical phase facilitates meaningful comparisons across studies and aids in establishing its prognostic significance in AOPP [29].

Considerations for Interpreting NLR Values

High NLR values have emerged as prognostic indicators in cases of acute OP pesticide poisoning , signaling a heightened risk of severe poisoning and in-hospital mortality. Specific NLR cutoffs, such as >13 and >17, have demonstrated moderate predictive capability for these adverse outcomes [17]. NLR offers a straightforward and easily accessible inflammatory marker that aids risk stratification and informs therapeutic approaches in AOPP. Elevated NLR levels upon admission serve as independent predictors of poor prognosis, underscoring the significance of promptly identifying and monitoring patients exhibiting high NLR values [30]. While NLR shows promise as a prognostic marker in AOPP, its performance warrants comparison with other clinical parameters. Studies have examined NLR alongside factors like serum cholinesterase and creatinine levels to assess its predictive capacity and independence as a risk factor for severe poisoning and mortality [31]. Identified as >13 and >17, the optimal cutoff values for NLR in predicting AOPP outcomes, including severe poisoning and in-hospital mortality, offer valuable insights into its discriminatory ability. Understanding these cutoff values and corresponding sensitivity and specificity enhances the interpretation of NLR values in AOPP. This enables healthcare professionals to effectively utilize this inflammatory marker for risk assessment, patient management, and treatment optimization in cases of AOPP.

Clinical studies on NLR and OPP

Review of Studies Assessing NLR as a Predictor of Outcomes

The NLR has been under scrutiny as a potential prognostic indicator in diverse cancers, including parotid gland cancer [32], rectal cancer [33], and colon cancer [34]. A high NLR may signify a systemic inflammatory response and oxidative stress, both implicated in cancer progression and metastasis [32,33]. Numerous studies have linked elevated NLR levels with poorer cancer-specific survival (CSS) and disease-free survival (DFS) compared to lower NLR levels [32,33,35]. For instance, in parotid gland cancer, patients with an NLR > 0.32 exhibited significantly worse CSS than those with an NLR ≤ 0.32 [32]. Similarly, in head and neck cancer, individuals with an NLR above the median faced a 2.69 hazard ratio (HR) for locoregional recurrence and a 1.74 HR for any recurrence or death compared to those with a lower NLR [35]. Optimal cutoff values for delineating high versus low NLR vary across studies, with some employing a single cutoff [33], while others utilize multiple risk categories [32,33]. Neoadjuvant therapy and lymph node yield did not exert significant effects on the prognostic value of NLR [33]. Evidence suggests that an elevated NLR independently predicts unfavorable survival outcomes in various cancers. Nevertheless, further research is warranted to standardize the definition of high NLR and validate its utility in larger prospective cohorts. Integrating NLR into risk stratification models could prove instrumental in guiding treatment decisions and determining follow-up intensity for high-risk patients.

Correlation Between NLR and Severity of Poisoning

In a study examining the severity and clinical outcomes of poisoning incidents, it was discovered that patients predicted to have severe illness based on severity scores were more prone to experiencing adverse outcomes, such as severe morbidity or death [36]. This study underscored a moderate correlation between the Glasgow Coma Scale (GCS) and the poisoning severity score (PSS), suggesting the potential of these scoring systems in predicting outcomes. Another study focused on carbon monoxide poisoning investigated the relationship between the PSS and carboxyhemoglobin (COHb) levels in affected patients [37]. The findings revealed a moderate correlation between PSS and outcome, with severe PSS grades significantly associated with mortality and major complications. The PSS is a standardized scale for grading the severity of poisoning incidents, enabling a qualitative assessment of morbidity resulting from poisoning and improved identification of risks [38]. PSS categorizes poisoning severity into grades ranging from NONE (0) to FATAL (4), based on observed clinical symptoms and signs, without considering parameters like ingested amounts or serum concentrations. These studies suggest that alongside established severity scoring systems like PSS and GCS, the NLR can also play a crucial role in predicting the severity and outcomes of poisoning incidents. The correlation observed between NLR and poisoning severity highlights the potential of this biomarker in risk stratification and guiding clinical management decisions.

NLR as a Prognostic Marker for Mortality and Morbidity

The NLR has garnered attention as a prognostic marker for mortality and morbidity across various medical conditions, including cancer. In the realm of breast cancer, research focusing on the lymph node ratio (LNR) has shown promising outcomes in predicting patient prognosis. Particularly in breast cancer patients with node-positive disease, the LNR has demonstrated superior predictive accuracy for survival compared to traditional pN staging methods, offering potential advantages in standardizing staging protocols and informing treatment decisions [39,17]. Studies have consistently revealed that a higher LNR is associated with poorer outcomes, with patients classified into high-risk LNR groups exhibiting lower rates of DFS and overall survival compared to those in low-risk groups [39]. Moreover, the LNR has emerged as an independent predictor of survival alongside traditional pN staging, underscoring its potential utility in risk stratification and treatment planning for individuals battling breast cancer. In the context of breast cancer, the NLR, mainly when considered alongside LNR, serves as a valuable prognostic marker for mortality and morbidity. Its incorporation into clinical practice provides clinicians with additional insights into patient outcomes, facilitating optimizing therapeutic strategies tailored to individual patient needs.

Clinical implications and future directions

Potential Use of NLR in Clinical Practice

Elevated NLR upon admission has emerged as a significant predictor of severity and mortality in cases of acute OPP, suggesting its potential utility in forecasting outcomes in this context [17]. In the hepatocellular carcinoma (HCC) prognosis, NLR has been recognized as a reliable, cost-effective, and noninvasive biomarker for assessing outcomes posttreatment. Generally, elevated pretreatment NLR indicates poor survival prospects in HCC patients. Its effectiveness is heightened when combined with other inflammation markers and considerations of the patient's clinical trajectory [39]. NLR has been explored as a potential marker for predicting early-onset delirium post-acute ischemic stroke (AIS). Patients exhibiting delirium upon admission showed significantly higher NLR values, suggesting a potential role for NLR in prognosticating delirium occurrence in AIS patients [17]. As a marker of systemic inflammation and immune response, NLR holds relevance across various medical conditions such as cardiovascular disorders, malignancies, diabetes mellitus, coronary artery disease, and inflammatory arthritis. It reflects the overall inflammatory status of the body and has demonstrated predictive capabilities in neurological and psychiatric conditions, including Alzheimer’s disease, Parkinson’s disease, and ischemic stroke [30]. In oncology, NLR finds utility in cancer stratification, correlating with tumor size, stage, metastatic potential, and lymphatic invasion. It independently predicts overall, cancer-free, and CSS. Additionally, NLR is a sensitive indicator of infection, inflammation, and sepsis, facilitating monitoring of oncological therapy and diagnosis of systemic infections [30]. NLR exhibits the potential for outcome prediction, inflammatory response monitoring, and prognostic assessment across diverse medical domains. Its simplicity, cost-effectiveness, and reliability render it a valuable tool in clinical practice, particularly when integrated with other markers and tailored to the patient's clinical context.

Integration of NLR with Existing Prognostic Markers

Research indicates that the NLR serves as an independent predictor of adverse prognosis in AOPP and can effectively aid in treatment optimization and patient management [31,17]. Elevated NLR levels have been linked with severe poisoning and increased in-hospital mortality rates, establishing it as a valuable prognostic marker [31]. Currently, prognostic markers utilized in AOPP encompass serum cholinesterase levels, creatinine levels, Acute Physiology and Chronic Health Evaluation II (APACHE II) scores, blood lactate levels, caspases, and other biochemical parameters, all of which contribute to predicting disease severity and outcomes [31]. However, NLR's straightforwardness, cost-effectiveness, and routine availability as part of standard blood tests position it as a promising addition to the existing prognostic armamentarium in AOPP [31]. Integrating NLR with these established markers can enhance risk stratification, inform therapeutic approaches, and ultimately enhance patient outcomes in cases of AOPP. By amalgamating NLR with established prognostic indicators in AOPP, a comprehensive approach can be achieved, facilitating a more thorough assessment of disease severity, outcome prediction, and optimization of treatment strategies for individuals afflicted with AOPP.

Areas for Further Research and Validation

To establish standardized NLR cutoffs for effective risk stratification, several studies propose thresholds such as 7.5, 13, and 17 to predict severity and mortality. However, further research must validate these cutoffs across diverse populations and settings. Larger prospective studies are needed to ascertain the optimal NLR cutoffs essential for informing clinical decision-making [31]. While a single elevated NLR upon admission has been associated with an unfavorable prognosis, the potential value of serial NLR measurements over time in monitoring disease progression and treatment response remains largely unexplored. Investigating the longitudinal trends of NLR could provide valuable insights into disease dynamics and treatment efficacy [31]. Comparative analyses between NLR and other prognostic markers reveal its superiority over inflammatory markers like C-reactive protein in predicting outcomes. However, further investigation is warranted to evaluate NLR's prognostic performance against established markers such as cholinesterase levels, APACHE II scores, and lactate levels. Such comparisons are crucial for determining NLR's relative utility and efficacy in prognostication [31]. Understanding the underlying mechanisms linking NLR to OP toxicity is essential for elucidating its role in disease severity and complications like the intermediate syndrome. While existing evidence suggests that elevated NLR reflects the inflammatory response to OPP, the precise pathophysiological mechanisms necessitate further exploration and clarification [26]. Considering the potential influence of confounding factors such as age, sex, and underlying conditions on NLR, future studies should meticulously account for these variables. Establishing the independent prognostic value of NLR in OPP requires robust analyses that control for potential confounders and delineate NLR's specific contribution to prognostication in this context [31].

## Conclusions

In conclusion, the NLR is a promising biomarker for predicting outcomes in AOPP. Throughout this review, we have delved into its role as a prognostic indicator, noting its association with the severity of poisoning and adverse clinical outcomes, including mortality and morbidity. Elevated NLR values offer valuable insights into the underlying inflammatory response triggered by OP toxicity, aiding in risk stratification and guiding clinical decision-making. Integrating NLR assessment into the clinical management of acute poisoning holds significant implications for improving patient outcomes. Early identification of high-risk patients based on NLR levels can facilitate prompt initiation of appropriate therapies, such as antidotal treatment and supportive care measures. Moreover, serial monitoring of NLR levels may assist in assessing treatment response and guiding the intensity of therapeutic interventions. However, further research efforts are warranted to validate the utility of NLR in larger patient cohorts and standardize its incorporation into clinical practice guidelines. Leveraging NLR as a prognostic tool has the potential to enhance risk stratification, optimize treatment strategies, and ultimately improve patient outcomes in the management of AOPP.

## References

[1] Robb EL, Regina AC, Baker MB (2024). Organophosphate Toxicity. https://pubmed.ncbi.nlm.nih.gov/29261901/.

[2] Patel A, Chavan G, Nagpal AK (2024). Navigating the neurological abyss: a comprehensive review of organophosphate poisoning complications. Cureus.

[3] (2024). What to know about organophosphate poisoning. https://www.medicalnewstoday.com/articles/320350.

[4] Asgarzadeh S, Ebadi A, Shahrbabaki AS, Safari S, Aghili SH, Farhang Ranjbar M, Sadeghi S (2024). National early warning score in predicting adverse outcomes for patients admitted to emergency department; a prognostic accuracy study. Arch Acad Emerg Med.

[5] Prabawa IP, Bhargah A, Liwang F (2019). Pretreatment neutrophil-to-lymphocyte ratio (NLR) and platelet-to-lymphocyte ratio (PER) as a predictive value of hematological markers in cervical cancer. Asian Pac J Cancer Prev.

[6] Fukuto TR (1990). Mechanism of action of organophosphorus and carbamate insecticides. Environ Health Perspect.

[7] Van Asperen K (1958). Mode of action of organophosphorus insecticides. Nature.

[8] (2024). Stress effects on the body. https://www.apa.org/topics/stress/body.

[9] (2024). Physiological Effects of Exercise | Changes & Benefits. https://study.com/academy/lesson/physiological-effects-of-physical-activity.html.

[10] Burton DA, Stokes K, Hall GM (2004). Physiological effects of exercise. BJA Education.

[11] Peter JV, Sudarsan TI, Moran JL (2014). Clinical features of organophosphate poisoning: a review of different classification systems and approaches. Indian J Crit Care Med.

[12] (2024). Organophosphate Toxicity Clinical Presentation. https://emedicine.medscape.com/article/167726-clinical.

[13] Oh SJ (2011). 18 - Treatment and management of disorders of the neuromuscular junction. Neuromuscular Disorders: Treatment and Management.

[14] Chakraborty RK, Burns B (2024). Systemic Inflammatory Response Syndrome. https://www.ncbi.nlm.nih.gov/books/NBK547669/.

[15] Camacho-Pérez MR, Covantes-Rosales CE, Toledo-Ibarra GA, Mercado-Salgado U, Ponce-Regalado MD, Díaz-Resendiz KJ, Girón-Pérez MI (2022). Organophosphorus pesticides as modulating substances of inflammation through the cholinergic pathway. Int J Mol Sci.

[16] Pradeep U, Mahajan DS (2024). A cross-sectional study of neutrophil to lymphocyte ratio as a prognostic marker in acute organophosphorus poisoning in a tertiary care hospital in Central India. F1000Res.

[17] Mu Y, Hu B, Gao N, Pang L (2021). Prognostic value of the neutrophil-to-lymphocyte ratio in acute organophosphorus pesticide poisoning. Open Life Sci.

[18] Dundar ZD, Ergin M, Koylu R, Ozer R, Cander B, Gunaydin YK (2014). Neutrophil-lymphocyte ratio in patients with pesticide poisoning. J Emerg Med.

[19] Ramadori GP (2023). Acute respiratory distress syndrome (ARDS) and cardiac failure as cause of death in hospitalized patients. Int J Mol Sci.

[20] Ruíz-Arias MA, Medina-Díaz IM, Bernal-Hernández YY (2023). Hematological indices as indicators of inflammation induced by exposure to pesticides. Environ Sci Pollut Res Int.

[21] (2024). Neutrophil to Lymphocyte Ratio (NLR), Complete Blood Count (CBC) With Differential and Platelet. https://www.labcorp.com/tests/005013/neutrophil-to-lymphocyte-ratio-nlr-complete-blood-count-cbc-with-differential-and-platelet.

[22] Khare S, Radian AD, Dorfleutner A, Stehlik C (2016). Measuring NLR oligomerization I: size exclusion chromatography, co-immunoprecipitation, and cross-Linking. Methods Mol Biol.

[23] Ahuja H, Mathai AS, Pannu A, Arora R (2015). Acute poisonings admitted to a tertiary level intensive care unit in Northern India: patient profile and outcomes. J Clin Diagn Res.

[24] (2024). Questions and Answers About Ricin. https://emergency.cdc.gov/agent/ricin/qa.asp.

[25] Banerjee I, Tripathi SK, Roy AS (2014). Clinico-epidemiological profile of poisoned patients in emergency department: a two and half year's single hospital experience. Int J Crit Illn Inj Sci.

[26] Jeon TJ, Park JY (2017). Clinical significance of the neutrophil-lymphocyte ratio as an early predictive marker for adverse outcomes in patients with acute pancreatitis. World J Gastroenterol.

[27] Zahorec R (2021). Neutrophil-to-lymphocyte ratio, past, present and future perspectives. Bratisl Lek Listy.

[28] Jiang WM, Xu JF, Chen J, Li GL, Gao YF, Zhang Q, Chen YF (2022). Prediction of long-term survival outcome by lymph node ratio in patients of parotid gland cancer: a retrospective study. Front Surg.

[29] Karjol U, Jonnada P, Chandranath A, Cherukuru S (2020). Lymph node ratio as a prognostic marker in rectal cancer survival: a systematic review and meta-analysis. Cureus.

[30] Jiang C, Wang F, Guo G (2019). Metastatic lymph node ratio as a prognostic indicator in patients with stage IV colon cancer undergoing resection. J Cancer.

[31] Farrokhian N, Holcomb AJ, Dimon E (2022). Assessing prognostic value of quantitative neck dissection quality measures in patients with clinically node-negative oral cavity squamous cell carcinoma. JAMA Otolaryngol Head Neck Surg.

[32] Churi S, Ramesh M, Bhakta K, Chris J (2012). Prospective assessment of patterns, severity and clinical outcome of Indian poisoning incidents. Chem Pharm Bull (Tokyo).

[33] Cevik AA, Unluoglu I, Yanturali S, Kalkan S, Sahin A (2006). Interrelation between the poisoning severity score, carboxyhaemoglobin levels and in-hospital clinical course of carbon monoxide poisoning. Int J Clin Pract.

[34] Pach J, Persson H, Sancewicz-Pach K, Groszek B (1999). Comparison between the poisoning severity score and specific grading scales used at the Department of Clinical Toxicology in Krakow (Article in Polish). Przegl Lek.

[35] Solak M, Turkoz FP, Keskin O (2015). The lymph node ratio as an independent prognostic factor for non-metastatic node-positive breast cancer recurrence and mortality. J BUON.

[36] Jin ML, Gong Y, Pei YC, Ji P, Hu X, Shao ZM (2020). Modified lymph node ratio improves the prognostic predictive ability for breast cancer patients compared with other lymph node staging systems. Breast.

[37] Lisa MD, Pistelli M, Ballatore Z (2016). Potential role of PLR and NLR in clinical decision making in locally advanced breast cancer. Ann Oncol.

[38] Mouchli M, Reddy S, Gerrard M, Boardman L, Rubio M (2021). Usefulness of neutrophil-to-lymphocyte ratio (NLR) as a prognostic predictor after treatment of hepatocellular carcinoma." Review article. Ann Hepatol.

[39] Kotfis K, Bott-Olejnik M, Szylińska A, Rotter I (2019). Could neutrophil-to-lymphocyte ratio (NLR) serve as a potential marker for delirium prediction in patients with acute ischemic stroke? A prospective observational study. J Clin Med.

